# Integrated copy number and miRNA expression analysis in triple negative breast cancer of Latin American patients

**DOI:** 10.18632/oncotarget.27250

**Published:** 2019-10-22

**Authors:** Bruna M. Sugita, Silma R. Pereira, Rodrigo C. de Almeida, Mandeep Gill, Akanksha Mahajan, Anju Duttargi, Saurabh Kirolikar, Paolo Fadda, Rubens S. de Lima, Cicero A. Urban, Kepher Makambi, Subha Madhavan, Simina M. Boca, Yuriy Gusev, Iglenir J. Cavalli, Enilze M.S.F. Ribeiro, Luciane R. Cavalli

**Affiliations:** ^1^ Department of Genetics, Federal University of Paraná, Curitiba, PR, Brazil; ^2^ Faculdades Pequeno Príncipe, Instituto de Pesquisa Pelé Pequeno Príncipe, Curitiba, PR, Brazil; ^3^ Department of Biology, Federal University of Maranhão, São Luis, MA, Brazil; ^4^ Department of Biomedical Data Sciences, Section Molecular Epidemiology, Leiden University Medical Center, Leiden, Netherlands; ^5^ Department of Oncology, Lombardi Comprehensive Cancer Center, Georgetown University Medical Center, Washington DC, USA; ^6^ Genomics Shared Resource, Comprehensive Cancer Center, The Ohio State University, Columbus, OH, USA; ^7^ Breast Unit, Hospital Nossa Senhora das Graças, Curitiba, PR, Brazil; ^8^ Department of Biostatistics, Bioinformatics, and Biomathematics, Georgetown University Medical Center, Washington DC, USA; ^9^ Innovation Center for Biomedical Informatics (ICBI), Lombardi Comprehensive Cancer Center, Georgetown University Medical Center, Washington DC, USA

**Keywords:** microRNA, triple-negative breast cancer, copy number, latinas, disparities

## Abstract

Triple negative breast cancer (TNBC), a clinically aggressive breast cancer subtype, affects 15–35% of women from Latin America. Using an approach of direct integration of copy number and global miRNA profiling data, performed simultaneously in the same tumor specimens, we identified a panel of 17 miRNAs specifically associated with TNBC of ancestrally characterized patients from Latin America, Brazil. This panel was differentially expressed between the TNBC and non-TNBC subtypes studied (*p* ≤ 0.05, FDR ≤ 0.25), with their expression levels concordant with the patterns of copy number alterations (CNAs), present mostly frequent at 8q21.3-q24.3, 3q24-29, 6p25.3-p12.2, 1q21.1-q44, 5q11.1-q22.1, 11p13-p11.2, 13q12.11-q14.3, 17q24.2-q25.3 and Xp22.33-p11.21. The combined 17 miRNAs presented a high power (AUC = 0.953 (0.78–0.99);95% CI) in discriminating between the TNBC and non-TNBC subtypes of the patients studied. In addition, the expression of 14 and 15 of the 17miRNAs was significantly associated with tumor subtype when adjusted for tumor stage and grade, respectively. In conclusion, the panel of miRNAs identified demonstrated the impact of CNAs in miRNA expression levels and identified miRNA target genes potentially affected by both CNAs and miRNA deregulation. These targets, involved in critical signaling pathways and biological functions associated specifically with the TNBC transcriptome of Latina patients, can provide biological insights into the observed differences in the TNBC clinical outcome among racial/ethnic groups, taking into consideration their genetic ancestry.

## INTRODUCTION

Breast cancer is the leading cause of death in women from Latin America living in the US or in Latin American countries (referred here as Latinas). The overall incidence and mortality rates of breast cancer in this population in the US were 91.9 and 14.0 per 100,000 in 2015 [1, 2]. In Brazil, 59,700 new cases of breast cancer were expected in 2018 [3]. The highest mortality rates were observed among Puerto Ricans (19.04), Mexicans (18.78) and Cubans (17.89), and the lowest rate was observed among Central and South Americans (10.15) [4]. The breast cancer 5-year survival rate in Latin America hardly exceeds 70%, which is usually correlated with late diagnosis; approximately 30%–40% of breast cancer patients in the Latin American countries, including Brazil, are diagnosed in stages III and IV of the disease [5].

There is a well-documented disparity of breast cancer in Latinas when compared to non-Hispanic Whites (NHW); Latinas are more likely to present with non-localized disease, receive less aggressive therapy, and have a disproportionately low survival rate when compared to NHW [6–8]. Several factors can contribute to the increased mortality rate in this population group, including socio-economic barriers, which can limit their overall access to early detection and cancer prevention services [9, 10], and presence of co-morbidities, such as obesity and diabetes [11–16]. Many deaths in this population can also be attributed to after-diagnosis factors, such as inadequate access to appropriate treatment or early treatment interruption or discontinuation [17–19].

Epidemiological and molecular studies have shown that breast cancer subtypes are distributed unevenly among various racial/ethnic groups [6, 7, 20–22]. The incidence of the triple negative breast cancer (TNBC) subtype in particular, one of the most clinically aggressive breast cancer subtypes, largely varies according to ethnicity; it is present at higher frequencies in African-American (AA) (24–42%) and Hispanic/Latina (15–33%) women when compared to NHWs (11–28%) [6, 23, 24]. As in AA women, Latina patients with TNBC are more often diagnosed at an earlier age, with advanced stage, likely to experience metastasis and be refractory to treatment [25–29]. In addition to the general attributed socio-economic factors mentioned above, tumor biology and genetic background play a significant role in such disparity [11, 14, 30–33], however the individual contribution of each of these factors to their observed poor clinical outcome remains unclear. There is also lack of basic science studies and clinical trials that are conducted in Latinas [34, 35] which limits the knowledge of the biological causes that contributes to these tumors’ clinical aggressiveness.

MicroRNAs (miRNAs) are a class of non-coding endogenous RNA molecules that have been identified to play a role in breast tumorigenesis [36, 37]. MiRNA expression has been shown to present different expression patterns according to the intrinsic molecular breast cancer subtype [38, 39]. In TNBC, for instance, distinct miRNAs were reported mediating cellular processes associated with aggressive tumor phenotypes, such as the ones that promote metastatic development [40–43] and treatment resistance [44, 45]. Interestingly, miRNAs were also shown to present variable expression according to race and/or ethnicity [46–50]; a number of studies have shown miRNA polymorphisms in association with the susceptibility risk of breast cancer in specific ethnic populations [49, 51–54]. Data on somatic miRNA expression levels in the breast tissue of these populations, are however, scarce [50, 55].

Although the characterization of the genomic profiles in TNBC has been extensively performed, few studies have characterized them in specific ethnic groups, such as Latinas. This translates to a deficiency in the understanding of the intrinsic characteristics of their tumors’ genome, which can differentially impact their tumor phenotypes and clinical behavior. Therefore, in this study our main aim was to determine the patterns of DNA copy number and miRNA expression of TNBC of ancestrally genomic characterized patients living in Latin America, Brazil. In addition, we aimed to determine whether copy number alterations (CNAs) could impact miRNA expression levels and whether there were common miRNA target genes affected by both CNAs and miRNA deregulation.

A distinct pattern of CNAs and miRNA expression was observed between the TNBC and non-TNBC cases analysed; TNBC cases presented a higher number of CNAs when compared to the non-TNBC, affecting distinct chromosome cytobands. The integrated analysis of CNAs and miRNA expression revealed 17 miRNAs differentially expressed between these subtypes, with their expression levels concordant with the patterns of the CNAs. This 17miRNA panel presented a high combined power in discriminating the TNBC and non-TNBC subtypes and most of these miRNAs were significantly associated with these subtypes when adjusting for tumor stage and grade. MiRNA target and functional enrichment analysis showed the involvement of these miRNAs in specific cancer signaling pathways, which can distincly impact the biological phenotype and clinical outcome of the TNBC patients studied.

## RESULTS

### DNA copy number profiling

DNA copy number analysis was performed by array-CGH for 25 cases of TNBC and 16 cases of non-TNBC subtypes of the patients studied. A total number of 292 and 204 CNAs (as measured by the “number of calls” per the aberration interval base reports (Agilent-CytoGenomics v.3.0) were identified in the TNBC and non-TNBC subtypes, with an average of 18.25±5.98 and 14.57±3.09 CNAs per case, respectively. These differences were not significant at *p <* 0.05 (Unpaired *t* test; *p* = 0.5902).

In the TNBC cases, 64.0% of the cases (16/25) showed significant CNAs; the most frequent cytobands affected were: 8q21.3-q24.3 (43.8% of the cases), 3q24-29 and 6p25.3-p12.2 (37.5% of the cases), 1q21.1-q44, 5q11.1-q22.1, 11p13-p11.2, 13q12.11-q14.3, 17q24.2-q25.3 and Xp22.33-p11.21 (31.3% of the case) and 10q23.31-q26.3, 12p13.33-p13.1, 14q21.1-q32.32, 15q26.1-q26.3, 16q11.2-q22.1 and 19p13.11-p12 (25% of the cases) (Table 1). Sixty-percent of these cytobands were affected by gains of copy number and 40% by losses. The identification of the genes mapped in these cytobands, per the aberration interval base reports, revealed a total number of 4,585 genes (ranging from 51 to 989 genes per cytoband). In the non-TNBC cases, 87.5% of the cases (14/16) showed significant CNAs, and the most frequent cytobands affected were 1q21.11-q44 (64.3% of the cases), 8q21.13-q24.3 (50.0% of the cases), 7q11.21-q36.3 (42.9% of the cases), followed by 6p22.3-p21.2, 17q21.32-q25.3 and 20q13.31-q13.33 (35.0% of the cases) and 19p13.11 (28.57% of the cases) (Table 1). In these cases, only gains of copy number were observed. A total of 3,057 genes (ranging from 324 to 1029 genes per cytoband) were found located in these affected cytobands. The combined array-CGH profiling of the TNBC and non-TNBC cases are presented in Supplementary Figure 1.

**Table 1:** Most common cytobands affected by CNAs and corresponding number of genes located in these regions observed in the TNBC and non-TNBC groups of patients analyzed (presented by chromosome numerical order)

TNBC						
Chr	Cytoband	Start	Stop	CNA	Cases (%)	# Genes*
chr1	q21.1 - q44	144988715	247737874	gain	5 (31.25%)	989
chr3	q24 - q29	148802495	194903188	gain	6 (37.50%)	242
chr5	q11.1 - q22.1	49690172	111370979	loss	5 (31.25%)	245
chr6	p25.3 - p12.2	2117686	52103799	gain	6 (37.50%)	609
chr8	q21.3 - q24.3	88884192	146066584	gain	7 (43.75%)	289
chr10	q23.31 - q26.3	89507004	135372492	loss	4 (25.00%)	360
chr11	p13 - p11.2	34322106	46565735	gain	5 (31.25%)	51
chr12	p13.33 - p13.1	309062	14132896	gain	4 (25.00%)	205
chr13	q12.11 - q14.3	19703703	53876286	loss	5 (31.25%)	216
chr14	q21.1 - q32.32	38723471	103447263	loss	4 (25.00%)	485
chr15	q26.1 - q26.3	90276459	102241406	gain	4 (25.00%)	63
chr16	q11.2 - q22.1	46693731	67933130	loss	4 (25.00%)	169
chr17	q24.2 - q25.3	65989022	80993001	gain	5 (31.25%)	232
chr19	p13.11 - p12	18266482	21108358	gain	4 (25.00%)	66
chrX	p22.33 - p11.21	1314894	58051765	loss	5 (31.25%)	364
**Non-TNBC**						
**Chr**	**Cytoband**	**Start**	**Stop**	**CNA**	**Cases (%)**	# Genes^*^
chr1	q21.1 - q44	145103876	249118400	gain	9 (64.28%)	1029
chr6	p22.3 - p21.1	18093033	43409896	gain	5 (35.71%)	458
chr7	q11.21 - q36.3	62516153	158909738	gain	6 (42.86%)	687
chr8	q21.13 - q24.3	82193925	146280020	gain	7 (50.00%)	324
chr17	q21.32 - q25.3	46048958	81029941	gain	5 (35.71%)	445
chr19	p13.11	17845278	17927374	gain	4 (28.57%)	4
chr20	q13.31 - q13.33	55212094	62893189	gain	5 (35.71%)	114

^*^cytobands locations, positions, size and # genes and miRNAs affected by CNAs based on the aberration interval base reports (Agilent CytoGenomics v. 5.0)

### Global miRNA expression profiling

**Figure 1:**
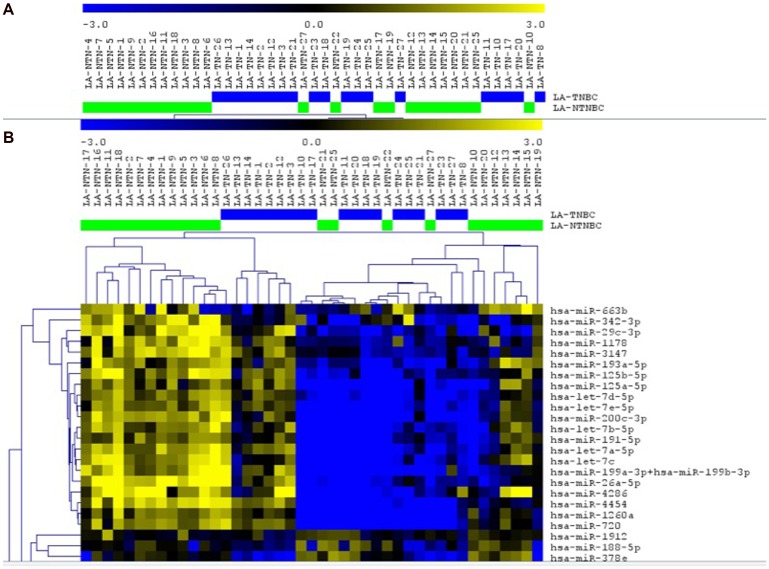
Unsupervised (**A**) and supervised (**B**) hierarchical clustering analysis applied to the TNBC (blue bars) and non-TNBC (green bars) cases analyzed. Up-regulated miRNAs (yellow) and down-regulated miRNAs (blue). Selected area of the heatmap showed. (MeV 4.9, Pearson Correlation, *p* < 0.05).

Global miRNA expression profiling was performed using the Nanostring technology in 19 and 24 cases of the TNBC and non-TNBC subtypes, respectively. A number of 163 miRNAs were identified with significantly differentially expression between these two groups (*p* ≤ 0.05, FDR ≤ 0.25) (Supplementary Table 1). Unsupervised (UHC) and supervised hierarchical clustering (SHC) analysis of these miRNAs showed a more concise cluster of the TNBC cases, while most of the non-TNBC were interspersed (Figure 1). Of the 163 miRNAs, 87 (53.4%) showed increased expression and 76 (46.6%) showed decreased expression in TNBC when compared to the non-TNBC cases. The miRNAs that presented with the highest changes (log2FC>2) in expression between these groups were: miR-187-3p, miR-601, miR-663a, miR-421, miR-378b and miR-1305. These six miRNAs were up-regulated in the TNBC group. In additional, 10 miRNAs (miR-720, miR-1260a, miR-4286, miR-4454, miR-200c-3p, let7b-5p, miR-199a-3p, miR-199b-3p, let-7c and let-7a-5p) were down-regulated in the TNBC group (Supplementary Table 2). To identify the function of each of the 163 identified differentially expressed miRNAs, DIANA miRPath analysis was used to perform pathway enrichment analysis (KEGG pathways). Among the top 10 pathways identified, based on the most significant adjusted *P* value (FDR corrected), were the ones related to ECM-receptor interaction, adherens junction, mucin type-O-glycan biosynthesis, morphine addiction and proteoglycans in cancer (Supplementary Table 3).

### Integration of miRNA and copy number alterations (CNAs) analysis

#### Mapping of miRNAs in cytobands affected by CNAs

**Figure 2:**
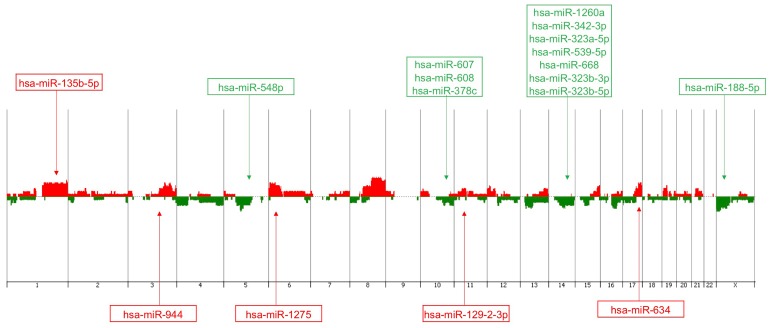
Genomic view/penetrance plot of the array-CGH profiling of the TNBC cases (*n* = 25) from the patients analyzed with the corresponding mapping of the 17 miRNAs of the identified panel. Vertical lines represent chromosome number. Red peaks indicate copy number gains and green peaks indicate copy number losses. The miRNAs expression levels, up- and down regulated, are represented in red and green color boxes, respectively (Agilent Genomic Workbench 7.0).

**Figure 3:**
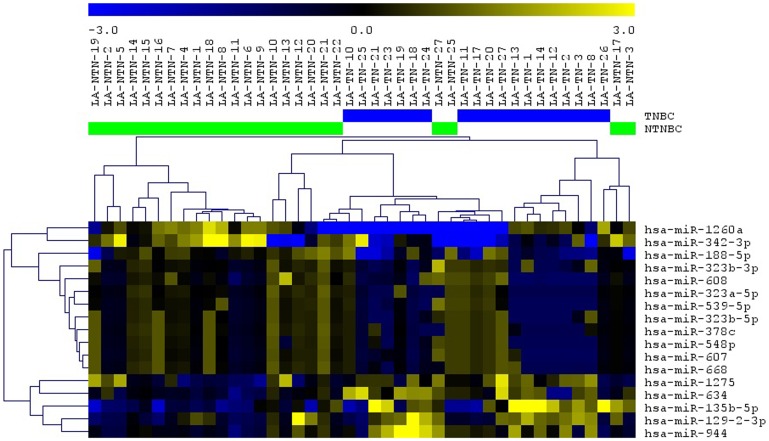
Unsupervised hierarchical clustering (UHS) analysis applied to the TNBC (blue bars) and non-TNBC (green bars) cases analyzed using the selected 17 miRNAs. Up-regulated and down-regulated miRNAs are represented in yellow and blue color, respectively (MeV 4.9, Pearson Correlation).

In order to identify miRNAs that could be affected by CNAs in the TNBC cases analyzed, the genomic location of the initial set of 163 miRNAs found to be differentially expressed between the TNBC and non-TNBC subtypes was verified. Forty-five of them (27.6%) were located in the cytobands mostly affected by CNAs in the same TNBC cases profiled by array-CGH as described above. From these 45 miRNAs, 17 (37.8%) presented expression levels in concordance with the observed CNAs at their respective genome *locus* (i. e. cytoband with gains/amplifications of copy number/up-regulated miRNA expression and/or losses/deletions of copy number/down-regulated miRNA expression) (Table 2, Figure 2). The analysis of each individual case per subtype, showed that in the TNBC group, 3 to 12 of the selected 17 miRNAs were observed with altered expression levels, with an average of 6.32 ± 0.525 miRNAs with alterations per case. In the non-TNBC group, 0 to 10 of these miRNAs presented expression alterations, with an average of 3.58±0.51 miRNAs with alterations per case. This difference was statistically significant (*p* = 0.0006), showing that in each of the TNBC cases there was a higher number of miRNA expression alteration levels of the selected 17 miRNA panel when compared to each of the non-TNBC cases. Unsupervised hierarchical clustering (UHC) analysis using expression levels of these miRNAs was able to distinctly cluster all the TNBC cases and most (exception of 4 cases) of the non-TNBC cases (Figure 3).

**Table 2:** Chromosome location of the seventeen differentially expressed miRNAs between the TNBC and non-TNBC groups of patients, with expression levels in concordance with copy number alterations (CNAs) (presented by chromosome numerical order)

miRNAs	Cytoband	Start	Stop	CNA	miRNA expression	Log2FC	Adj p
hsa-miR-135b-5p	1q32.1	205448302	205448398	gain	up-regulated	1.93	0.001
hsa-miR-944	3q28	189829922	189830009	gain	up-regulated	1.13	0.004
hsa-miR-548p	5q21.1	100816482	100816565	loss	down-regulated	−0.53	0.002
hsa-miR-1275	6p21.31	33999972	34000051	gain	up-regulated	0.61	0.049
hsa-miR-607	10q24.1	96828669	96828764	loss	down-regulated	−0.50	0.006
hsa-miR-608	10q24.31	100974985	100975084	loss	down-regulated	−0.52	0.04
hsa-miR-378c	10q26.3	130962588	130962668	loss	down-regulated	−0.42	0.024
hsa-miR-129-2-3p	11p11.2	43581394	43581483	gain	up-regulated	0.77	0.036
hsa-miR-1260a	14q24.3	77266218	77266290	loss	down-regulated	−3.30	0.004
hsa-miR-342-3p	14q32.2	100109655	100109753	loss	down-regulated	−1.51	0.039
hsa-miR-323a-5p	14q32.31	101025732	101025817	loss	down-regulated	−0.59	0.001
hsa-miR-539-5p	14q32.31	101047321	101047398	loss	down-regulated	−0.68	<0.05
hsa-miR-668	14q32.31	101055258	101055323	loss	down-regulated	−0.45	0.014
hsa-miR-323b-3p	14q32.31	101056219	101056300	loss	down-regulated	−0.49	0.008
hsa-miR-323b-5p	14q32.31	101056219	101056300	loss	down-regulated	−0.49	0.009
hsa-miR-634	17q24.2	66787072	66787168	gain	up-regulated	0.90	0.005
hsa-miR-188-5p	Xp11.23	50003503	50003588	loss	down-regulated	−0.93	0.02

#### Identification of miRNA target genes with concomitant miRNA expression and CNAs

In addition to the mapping of the miRNAs in the cytobands affected by CNAs as described above, we then searched for the miRNA gene targets that could be potentially affected by the regulation of the 17 miRNAs mapped in these regions. Using miRNA target prediction databases, a total of 10,675 targets were identified, as predicted from more than two independent databases. MiR-608, mapped at 10q24.31, was the miRNA with the highest number of gene targets (5,268 genes) and miR-129-2-3p, mapped at 11p11.2, with the lowest (131 genes). Next, these miRNA targets were matched with the genes that were mapped in the cytobands affected by CNAs, as generated by the aberration interval base report (array-CGH gene list) (4,585 genes). The integration of these data revealed 2,098 common genes (Supplementary Figure 2), i. e. genes that could be potentially affected by both CNAs and miRNA expression deregulation. This integration approach reduced the initial number of the total miRNA targets by 80.3% (from 10,675 to 2,098).

#### Biological function and pathway enrichment analysis of the miRNAs and corresponding gene targets affected by miRNA expression and CNAs

In order to identify the main biological function of the 17 selected miRNAs, we performed pathway enrichment analysis (KEGG pathways) using Diana miRPath v.3.0. Two major miRNAs clusters among the 17 miRNAs regulating these pathways were found: one formed by four miRNAs (miR-539, miR-548, miR-607 and miR-944) and the other by thirteen miRNAs (miR-129-2-3p, miR-135b-5p, miR-188-5p, miR-323a-5p, miR-323b-3p, miR-323b-5p, miR-342-3p, miR-378c, miR-608, miR-634, miR-668-3p, miR-1260a and miR-1275) (Supplementary Figure 3). The top 10 pathways identified involving these miRNAs were the axon guidance (hsa04360), glycosaminoglycan biosynthesis-chondroitin sulfate/dermatan sulfate (hsa00532), mucin type-O-Glycan biosynthesis (hsa00512), thyroid hormone signaling pathway (hsa04919), signaling pathways regulating pluripotency of stem cells (hsa04550), proteoglycans in cancer (hsa05205), wnt signaling pathway (hsa04310), hippo signaling pathway (hsa04390), ras signaling pathway (hsa0414) and pathways in cancer (hsa05200). Remarkably, all the 17 selected miRNAs were presented in four of the top 10 pathways identified. The less representative pathways by these miRNAs were the mucin type-O-glycan biosynthesis and the glycosaminoglycan biosynthesis-chondroitin sulfate/dermatan sulfate, with the involvement of 29.4% and 41.2% of the miRNAs, respectively (Supplementary Table 4).

Identification of KEGG pathways potentially affected by the 17 miRNAs were also conducted considering only the 2,098 putative target genes presented in the cytobands with CNAs previously selected in the integrated analysis above. As a result, three KEGG pathways were found to be affected by ten out of the 17 miRNA panel: glycosaminoglycan biosynthesis - chondroitin sulfate/dermatan sulfate (hsa00532, *p* = 0.01967), biosynthesis of unsaturated fatty acids (hsa01040, *p* = 0.01967) and hippo signaling pathway (hsa04390, *p* = 0.01967) (Supplementary Table 5). Eight miRNAs of 17 miRNA panel were involved in the hippo signaling pathway: three upregulated (miR-944, miR-135b-5p and miR-1275) and five downregulated (miR-548p, miR-607, miR-323b-3p, miR-342-3p and miR-539-5p). Using Cytoscape and Diana miRpath data (Supplementary Figure 4), we observed that these miRNAs can act by targeting key regulators of this pathway, potentially damaging their control over several other biological functions, e. g. miR-944 targets *CRB1, MPP5* and *WWTR1* genes, which are involved in tight and adherens cell junction. Interestingly, three out of the four downregulated miRs (miR-548p, miR-323b-3p and miR-607) were shown to target *CCND2* gene, a cell cycle regulator.

**Figure 4:**
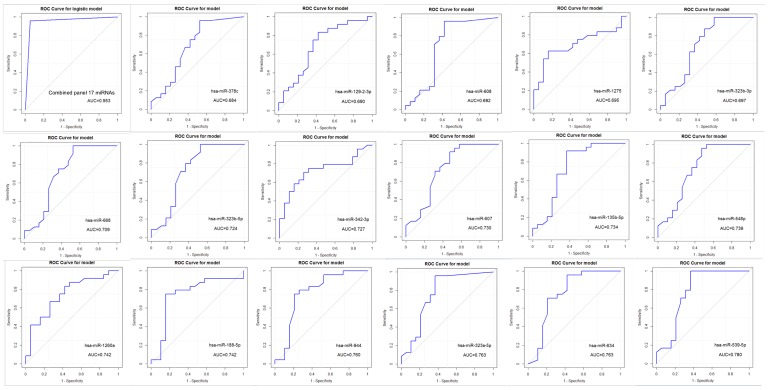
Receiver operating characteristic (ROC) curve plots and Area Under the Curve (AUC) values of the combined (top left) and individual 17 miRNAs differentially expressed between the TNBC and non-TNBC groups of patients.

### Individual and combined discriminatory power of the 17 miRNA panel between the TNBC and non-TNBC groups of patients

Each miRNA composing the identified 17 miRNA panel was evaluated in relation to its power in discriminating the TNBC and non-TNBC subtypes of the Latina patients (Figure 4). ROC analysis showed that 70% (12/17 = 70.6%) of the miRNAs presented an Area Under the Curve (AUC) value higher than 0.7. The miR-539-5p, miR-634, miR-323a-5p, miR-944 and miR-1260a miRNAs presented the highest discriminatory power, with AUC values ranging from 0.742 (miR-1260a) to 0.780 (miR-539-5p). The combined analysis of the panel showed a AUC value of 0.953, demonstrating a robust power of the complete17 miRNA panel in discriminating the TNBC and non-TNBC subtypes of the cases studied.

### Association of miRNA expression with the clinical-pathological variables of the patients studied

The association of the miRNA expression data with clinical-pathological information of the patients was performed in 43 samples (19 TNBC and 24 non-TNBC) (Table 3). Given the retrospective nature of this study, there were substantial missing clinical data from 13 individuals, who were therefore not included in this analysis. The age and tumor size data were log-transformed in order to be more normal-like before applying the Student *t* test; neither variable was associated with TNBC status (*p* = 0.46 for age, *p* = 0.34 for tumor size). The categorical variables were assessed by Fisher’s exact test and the following results were obtained: histological type and tumor grade were not significantly different between the TNBC and non-TNBC groups (*p* = 0.12 and *p* = 0.06, respectively), while lymph node status and tumor stage were significantly different (*p* = 0.002 and *p* = 0.006, respectively).

**Table 3:** Clinical and histopathological information from TNBC and non-TNBC groups

	TNBC (*n* = 27)	non-TNBC (*n* = 27)	*P* value
Age	54.25 ± 3.59	58.15 ± 2.34	
	16–83	34–88	
	(*n* = 20)	(*n* = 27)	*p* = 0.46
Tumor size (cm)	3.10 ± 0.47	3.48 ± 0.39	
	0.9–10.5	0.7–8.0	
	(*n* = 23)	(*n* = 26)	*p* = 0.34
Grade			
I/II	12	21	
III	11	5	*p* = 0.06
Tumor Stage			
Well differentiated	2	2	
Moderately differentiated	11	20	
Poorly differentiated	12	4	*p* = 0.002
Lymph node			
Positive	10	21	
Negative	10	3	*p* = 0.006

Two linear regression models were considered for each of the 17 miRNAs selected for integration, with the log-transformed miRNA values as outcomes and the covariates: age, tumor size, tumor subtype, histological type, lymph node status, and either tumor stage or tumor grade for each model. Both complete case analyses and multiple imputation analyses were considered. Tumor stage and tumor grade were not included in the same model due to their high degree of overlap: of the 43 cases, 2 did not present information for both tumor stage and grade; for the remaining 41 samples, 25 were of tumor grade I or II and moderate or well differentiated tumor stage, while 14 were of tumor grade III and poorly differentiated stage. For the complete-case analyses, which included 30 cases, 15 of the 17 miRNAs presented significant associations with tumor subtype (at a FDR = 0.05) when considering tumor grade in the regression analysis and 14 miRNAs presented significant associations with tumor subtype when considering tumor stage; the non-significant miRNAs in these analysis were miR-1275 and miR-129-2-3p and miR-1275, miR-129-2-3p, and miR-378c, respectively. None of the other variables were significantly associated with the 17 miRNAs expression values. The multiple imputation results were very similar - 16 out of the 17 miRNAs had significant associations with tumor subtype (at a FDR = 0.05 using Benjamini-Hochberg) when considering either tumor grade or tumor stage in the regression analysis. In both cases, the non-significant miRNA was miR-1275 and no other variables presented significant associations with miRNA values. Furthermore, the direction of the associations always remained the same as in the univariate analyses between miRNA values and tumor type.

### Association of miRNA expression with survival using KMPlot database

**Figure 5:**
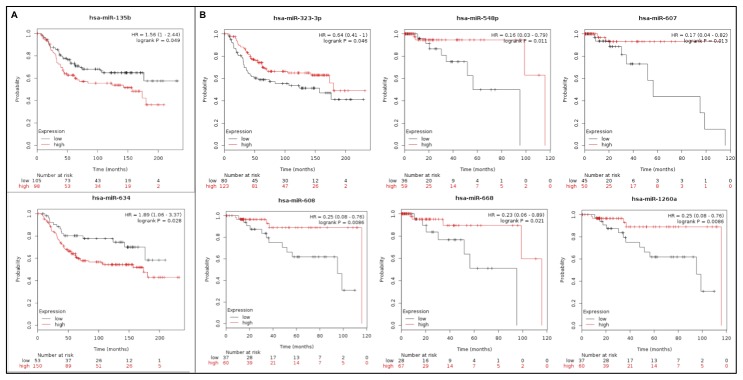
Kaplan–Meier plot results of eight out of the panel of 17 miRNAs that were differentially expressed between the TNBC and non-TNBC dataset of the METABRIC and TCGA data (KMPlot - miRpower). (**A**) Up-regulated miRNAs (**B**) Down-regulated miRNAs.

The miRNA expression levels of eight out of the 17 miRNAs in our panel were previously associated with TNBC survival in the analysis of the KMPlot datasets from the TCGA and METABRIC cohorts of basal-like/TNBC patients (Supplementary Table 6). In these TNBC cohorts, higher expression levels of miR-135b-5p and miR-634, as observed in our study, were significantly associated with reduced overall survival (OS) (months) **(** Figure 5A **).** Lower expression levels of miR-323b-3p, miR-548p, miR-607, miR-608, miR-668, and miR-1260a, was also observed in our study, were also associated with lower survival (Figure 5B).

## DISCUSSION

Population-based studies in North American patients, with breast cancer subtypes classified mostly by Immunohistochemistry (IHC) surrogate markers, have reported that Hispanic/Latina women are more likely to present with estrogen receptor negative (ER-) tumors, compared to non-Hispanic White (NHW) women [6, 26]. This data is similar to what has been reported among the distribution of the breast cancer subtypes in African American (AA) and NHW women [9, 56, 57]. Triple negative breast cancer (TNBC), an aggressive disease that rapidly progresses [58], has been shown to be more prevalent in Latina women (both living in US and in their countries of origin) (from 15 to 35%) compared to NHW women (from 8 to 15%) [24, 59]. However, the prevalence of these tumors greatly vary in the diverse Latin American countries, including Brazil [24]. In Brazil, the frequency of TNBC is about 17%, similar to the frequencies in Costa Rica and lower than the frequencies in Mexico, Peru and Colombia (from 20 to 35%) [59–63]. These frequency variations can be related to the differences in the subtype classification methods used, biases associated with clinic/hospital and/or registry based studies, lifestyle and environmental exposures and socio-economic factors [64, 65].

Increasing evidence has demonstrated that biological factors can account not only for the prevalence but for the higher mortality rate of TNBC that is observed in minority populations [21, 25, 50, 66–68]. In AA patients, several studies [21, 66], including our own [50], have shown differences in the tumor molecular profiles, both at the DNA copy number and miRNA expression level, when compared to the NHW patients. It has been shown, that in fact, gene expression profiles might change according to the genetic ancestry of an individual’s genome [67]. Characterization of the molecular profiles in each breast cancer subtype has been extensively performed, however, only a small number of studies have characterized them in specific ethnic groups, such as Latinas [21, 25, 68]. Considering the clinical implication of the molecularly defined subtypes, this lack of biological knowledge can directly impact the prognosis and treatment of this and other underrepresented populations.

MiRNA expression patterns have been shown, like gene expression patterns, to discriminate between the major breast cancer subytpes, in both cell lines and clinical studies [69, 70]. Race and ethnicity has also been shown to play a role in miRNA expression patterns; most of the data is however on germline miRNA alterations and their associated risk of breast cancer [71], rather than on somatic tumor cells.

In this study, using an integrated analysis of genome-wide copy number and miRNA profiling we identified a panel of 17 miRNAs differentially expressed between the TNBC and non-TNBC groups of patients that live in Latin America, specifically in the South of Brazil. Receiver Operating Characteristic (ROC) curve analysis showed a remarkable high power of this miRNA panel in discriminating the TNBC and non-TNBC subtypes of these groups.

In TNBC, five of these miRNAs were observed to be up-regulated (miR-135b-5p, miR-944, miR-1275, miR-129-2-3p and miR-634) and 12 down-regulated (miR-548p, miR-607, miR-608, miR-378c, miR-1260a, miR-342-3p, miR-323a-5p, miR-539-5p, miR-668, miR-323b-3p, miR-323b-5p and miR-188-5p) when compared to the non-TNBC group of patients. These miRNAs were selected among the significantly differentially expressed miRNAs among these subtypes and based on their localization in genomic regions affected by copy number alterations (gains/amplifications and/or losses/ deletions), as detected in the same TNBC specimens by array-CGH analysis. These regions involved mainly the 1q, 3q, 5q, 6p, 10q, 11p, 14q, 17q and Xp cytobands, some of which recurrently described as altered in TNBC [72].

All five observed up-regulated miRNAs were previously reported with altered expression in breast cancer studies. MiR-135b was found upregulated both in breast cancer cell lines and clinical cases, and shown to confer higher proliferation, migration and invasion activity in MCF-7, MDA-MB-231 and MDA-MB-468 cells [73–75]. These activities may be the result of miR-135b regulation of Wnt pathway by targeting the *APC* gene in MDA-MB-231 and MDA-MB-468 cell lines [74]. A similar oncogenic activity, in addition to promoting resistance to cisplatin, was shown for miR-944 in the MCF7 and MDA-MB-231 cells [76]. Higher expression of miR-135b-5p was also associated with ER-negative breast cancer [77, 78] and found to regulate ERα protein levels by interacting with its 3′UTR regions [75]. Interestingly, miR-135b-5p was found downregulated in metastatic microinvasive breast carcinoma samples when compared to non-metastatic microinvasive breast carcinoma samples and its suppression was shown to increase proliferation, migration and invasion capacity by targeting syndecan binding protein (*SDCBP*) gene [79]. These might suggest that miR-135b-5p may play different roles depending on tumor stage by targeting different set of genes: in initial stages, high levels of miR-135b-5p may be important for promoting the formation of tumor while in late stages its lower expression can induce invasion and metastatic activity. Differential expression of miR-135b may also contribute to TNBC molecular heterogeneity as higher levels of this miRNA were found in basal-like cases (EGFR and CK5/6 positivity) when compared to non-basal like cases. Expression levels of miR-135b were also significantly associate with Ki67 (β = 0.94, *p <* 0.05) and AR expression (β = −25.9, *p <* 0.05): overexpression of miR-135b was positively correlated with high Ki67 expression (ρ = 0.434, *p <* 0.05) and low levels of miR-135b showed a negative correlation with AR expression (ρ = −0.276, *p <* 0.05) [80]. Other studies have also reported higher levels of miR-135b in basal-like tumor subtypes [81, 82] and suggests a correlation between its overexpression and poor survival and early metastasis relapse [83]. Upregulation of miR-1275 levels was particularly found in breast cancer of young women (<35 years) when compared to older women [84] and was also found hypomethylated in samples of healthy individuals who developed breast cancer when compared to individuals who remained healthy [85]. Although this combined evidence warrants further validation, it suggests that this miRNA might play a role in the initiation of breast cancer and could be used as a predictive cancer biomarker. MiR-129-2, that targets the Progesterone Receptor (PR) gene, has been described as upregulated in patients with low PR expression levels (PR-) [86]. This target suppression, is compatible with our findings, showing the up-regulation of miR-129-2 in the TNBC cases when compared to the non-TNBC cases. Finally, miR-634 was shown to regulate HER2 signaling by inducing apoptosis and inhibiting levels of HER2, p-AKT and p-ERK [87]. As TNBC have no expression of HER2, miR-634 may present a different role in these tumors by targeting other genes.

Among the 12 down-regulated miRNAs in our TNBC cases, miR-548p, miR-539-5p, miR-342-3p and miR-668 were previously reported with altered expression in breast cancer, with possible function as tumor suppressors [88–95]. The miR-548p’s anti-oncogenic activity was observed in two different studies in breast cancer; in the study of Shi *et al* (2015) its up-regulation was found to inhibit cell proliferation and induce apoptosis by targeting Enoyl Coenzyme A Hydratase short chain 1 (*ECHS1)* [88] and in the study of Ke *et al* (2016), it was found to perform the same functions by targeting the Nuclear Paraspeckle Assembly Transcript (*NEAT)* gene [89]. MiR-539-5p was also found to interfere with cell proliferation activity, in addition to migration, by regulating EGFR expression in MCF-7 and MDA-MB-231 cell lines [90] and lamin subunit alpha 4 (*LAMA4*) gene in BT549 cell line [91]. Low levels of miR-342-3p was reported in TNBC and is significantly associated with poor prognosis. Its tumor supressor activity was already shown in several TNBC cell lines (BT549, SUM149, SUM159, MDA-MB-157 and MDA-MB-468) as miR-342-3p can supress cell growth, viability and migration activity [92]. Expression of miR-342-3p is positively correlated with ERα [93] and its downexpression has been associated with tamoxifen resistant breast tumors [93, 94]. In ER-positive cases, high expression of miR-342 has been associated with better survival but not in ER-negative or TNBC cases, highlighting its role in tamoxifen response [95].

Expression levels of miR-1206a were already inversely correlated with HER-2 expression levels in MCF-7 and BT474 cell lines [96]. Up-regulation of miR-1206a was observed in HER-2 overexpressed MCF-7 cells when compared to regular MCF-7 cells, and downregulation of miR-1206a was observed in HER-2 intervened BT474 cells when compared to regular BT474 cells. These results corroborates our findings that miR-1260a was found down-regulated in TNBC cases. Interestingly, altered expression of miR-668 and miR-1206a may present an importance for non-TNBC patients as their overexpression presented clinical relevance in different studies. Overexpression of miR-668 was found to confer resistance to previously radiosensitive MCF-7 and T-47D cells by targeting Ikbα [97], and high levels of circulating miR-1260a in serum samples of metastatic breast cancer patients was related to poor prognosis [98]. Overexpression of miR-1260a was also found to be associated with poor prognosis in studies with different tumor types: melanome [99], neuroblastoma [100], prostate [101]. These findings suggest that mR-668 and miR-1260a present potential as prognostic biomarkers for non-TNBC patients.

Identification of the main pathways and networks potentially affected by the 17 miRNAs and putative gene targets resulted in a total of 46 KEGG pathways, among them Axon guidance, Glycosamin biosynthesis – chondroitin sufate/ dermatan sulfate and Mucin type O-Glycan biosynthesis. Interestingly, all of the 17 miRNAs of the identified panel were found to regulate gene targets associated with cancer signaling pathways, such as the Wnt, Ras, ErbB and Rap1. It is of note that 15 out of the 17 of the miRNAs were found to affect the Hippo signaling pathway.

A second integration analysis of the genes that were potentially affected by the CNAs (identified by the copy number profiling) and the corresponding gene targets of the 17 miRNAs located at the same affected cytobands, revealed a total of 2,098 genes mapped in these regions, suggesting that they may be commonly affected by both of these mechanisms. Among them were included genes that are critical to the TNBC tumorigenesis (and miRNA biogenesis), such as *CDKN1A, DICER1, ETV6, IGF1R, MYC* and *PIK3CA.* Enrichment functional analysis of these genes revealed three signaling pathways preferentially involved including the Hippo signaling pathway, observed in the first data integration approach presented above. Eight out of the 17 miRNAs (miR-1275, miR-135b-5p, miR-323b-3p, miR-342-3p, miR-5395p, miR-548p, miR-607 and miR-944) were found related to this pathway, potentially targeting 18 genes: *DVL3, PPP2RD2, LATS2, TCF7L2, CCND2, FZD6, WNT8B, BTRC, CSNK1D, FRMD6, STK3, YWHAZ, MPP5, SAV1, SOX2, CRB1, GDF6 and WWTR1.* A search on miRTarBase database however, showed that few of these interactions were experimentally validated [102]. Considering the critical role of the Hippo signaling pathway in regulating cell proliferation and apoptosis, and other tumorigenic processes, it is relevant to pursue downstream functional studies to confirm these interactions and determine their biological significance to the TNBC phenotype.

Finally, the association of the expression of the 17 miRNA panel with the clinical-histopathological parameters from the patients showed, except for three miRNAs, miR-1275, miR-129-2-3p, and miR-378c, association with tumor grade and tumor stage. Interestingly, eight out of the 17 miRNAs were previously associated with TNBC survival in the analysis of the KMPlot datasets from the TCGA and METABRIC cohorts of basal-like/TNBC patients. In particular, the up-regulation of miR-135b-5p and miR-634 and down regulation of miR-323b-3p, miR-548p, miR-607, miR-608, miR-669 and miR-1260a levels, were associated with reduced overall survival.

In conclusion, the integrated analysis of DNA copy number alterations and miRNAs expression levels, performed in this study, led to the identification of a robust 17 miRNA panel, with a high power in discriminating between the TNBC and non-TNBC subtypes of Latina patients. The clinical validation of this panel in a novel and independent ancestrally characterized Latina population, can reveal whether this panel, or a subset of its composing miRNAs, can represent the intrinsic biology of their TNBC transcriptomes, that can differentially impact their tumor phenotypes and clinical behavior.

## MATERIALS AND METHODS

### General study design

Genome-wide array-CGH and miRNA profiling were performed in TNBC and non-TNBC subtypes of patients from Latin America, Brazil, to detect the patterns of DNA copy number alterations (CNAs) and changes in miRNA expression, respectively. The differentially expressed miRNAs between the subtypes were integrated with copy number profiling data performed in the same TNBC tissue specimens, to select miRNAs that were mapped in regions affected by CNAs and gene targets potentially affected by both miRNA deregulation and CNAs. Combinatorial target prediction algorithms in conjunction with functional and pathway annotation enrichment systems were then applied to the selected miRNAs to identify the most relevant miRNAs and their corresponding targets associated with TNBC. Receiver Operating Characteristic (ROC) curve analysis was performed to determine the individual and combined power of the differentially expressed miRNAs in discriminating the TNBC and non-TNBC subtypes of the patients. Finally, the molecular data was associated with clinical-pathological information from the patients and external survival data to determine their potential prognostic relevance.

All experiments of this study were performed in accordance with relevant guidelines and regulations.

### Patient accrual and sample collection

Fifty-four formalin-fixed paraffin-embedded (FFPE) tissue samples of non-treated primary breast tumors were collected from the pathology tumor bank at the Hospital Nossa Senhora das Graças (HNSG), Paraná, Brazil. All samples were transferred to Georgetown University with no patient identifiers, under patient informed consent and through the IRB approval of Georgetown University, HNSG and the National Review Board of Ethics in Research (CONEP-Brazil). The TNBC and non-TNBC subtypes were determined by ER, PR and HER2 receptors status by immunohistochemistry (IHC) analysis performed at the time of diagnosis, following international guidelines [103, 104]. Briefly, the Monoclonal Mouse Anti-Human Estrogen Receptor α and Polyclonal Rabbit Anti-Human Progesterone Receptor were used for ER and PR analysis, respectively. ER and PR positivity were considered using a cut-off of 1%. The HercepTest (Dako North America Inc, Carpinteria, CA, USA) was used for HER2/Neu+ status. Using these criteria, 27 patients presented tumors of the TNBC subtype and 27 of the non-TNBC subtype (10 ER+/PR+/HER2-, 3 ER+/PR+/HER2+, 3 ER-/PR+/HER2-, 2 ER+/PR-/HER2- and 1 ER+/PR-/HER2+). In the remaining eight non-TNBC cases, 6 did not present information for PR expression (5 were ER+/HER2- and 1 ER+/HER2+), one for HER2 status (ER+/PR+) and one for ER and PR status (HER2+).

The clinical and histopathological information from the patients was retrieved in a de-codified manner from the pathology reports, and included age, tumor size, tumor stage and tumor grade and lymph node status (Table 3).

### DNA and RNA isolation

Prior to DNA and RNA isolation, the FFPE specimens were evaluated by the pathologist for the presence of at least 80% of tumor cells. The selected tumor areas were microdissected from unstained 10μm FFPE tissue sections and used for the subsequent molecular analysis. Consecutive tissue sections from the same tissue blocks were used to isolate DNA and RNA ensuring a direct correlation of DNA copy number and miRNA expression profiling data, as previously performed by our group [50].

DNA isolation was performed using phenol-chloroform protocol optimized for FFPE material [105] and RNA isolation was performed using MasterPure™ Complete DNA and RNA Purification kit (Epicentre Biotechnologies) following manufacture protocol. DNA and RNA quantity and quality were assessed using NanoDrop™ Spectrophotometer (Thermo Scientific Inc.) and the Bioanalyzer (Agilent Technologies Inc.), respectively.

### Ancestral analysis

The Latina population is highly heterogeneous and comprises individuals of several different genetic ancestries [106–109]. To obtain genomic based information of the population studied in relation to ethnicity, a subset of the patients (15 patients) was genotyped using the SNP chip Illumina Infinium QC Array (Illumina Inc., CA, USA), which contains 15,949 markers, including approximately 3,000 ancestral informative markers (AIMs). The genotype calling was performed as we previously described [50], using the GenomeStudio Software v. 2011.1. SNPs with MAF ≤ 0.01 were excluded from analysis. The data obtained was subsequently merged with the 1000 Genomes Project phase 1 (*n* = 1,902 samples) dataset [67], which present an overlap of 14,718 variants with the one from our study. Finally, Principal Components Analysis (PCA) was performed using PLINK 1.9 [110], which uses the EIGENSTRAT method [111] to calculate model ancestry differences between different samples. Based on the results of PC1 and PC2 (Supplementary Figure 5) the patients of this study clustered with the European (EUR) defined group from the 1000 Genome Project as well as with the Admixed Americans (AMR) main group, mainly composed of Colombians and Mexicans. This data was not surprising considering the markedly ancestral heterogeneity of the Brazilian population [112, 113].

### Array-CGH analysis

Genome-wide copy number profiling was performed by array-CGH using the SurePrint G3 Human CGH Microarray (Agilent, Santa Clara, CA, USA) according to our previous protocol for FFPE samples [105]. DNA isolated from peripheral blood from multiple normal individuals was used as control (reference) DNA. Control and case samples were directly labeled using the Bioprimer a-CGH Genomic Labeling kit and hybridized to the arrays for 40 hours. The arrays were scanned using Scanner Agilent G2565CA, and the data extracted using Feature Extraction (FE) software v10.10 (Agilent Tech. Inc.). The Agilent Cytogenomics v.3.0 software (Agilent Technologies Inc., Santa Clara, CA, USA), was used to analyze the data, using the algorithm ADM-2, threshold of 6.0 and an aberration filter with a minimum of 3 probes. Gene amplifications and deletions were defined as minimum average absolute log2 ratio (intensity of the Cy5 dye (reference DNA)/intensity of the Cy3 dye (test DNA) value of >0.25 and <-0.25, respectively, as per Agilent Cytogenomics guidelines. The number of “calls” (total significant number of CNAs) and the specifically affected cytobands were obtained from the generated aberration interval base reports (Agilent Cytogenomics v.3.0).

### MiRNA expression analysis

Global miRNA expression profiling was performed using NanoString nCounter technology Human v2 miRNA Expression Assay, as we previously described [50]. This specific assay contains 800 endogenous miRNAs, six positive miRNA assay controls, six negative miRNA assay controls, and five housekeeping transcripts (*ACTB, B2M, GAPDH, RPL19, RPLP0*). Raw miRNA expression data was pre-processed and normalized using NanoString’s nCounter RCC collector and nSolver v2 software respectively. Unsupervised (UHC) and supervised hierarchical cluster (SHC) analysis were performed on significantly differentially expressed miRNAs among the patients’ subtypes, using Pearson’s correlation coefficient, average linkage and Benjamini-Hochberg multiple testing correction on the Multiexperiment Viewer software (MeV 4.9.0) (*t*-test, *p <* 0.05, FDR ≤ 0.25). Fold changes, represented on the log2 scale (log2FC), were calculated for all differentially expressed miRNAs. Adjusted *p*-values were used to rank miRNAs of interest.

### Integrated analysis of array-CGH and miRNA data

Direct integration of the most differentially expressed miRNAs associated with the TNBC subtype with CNAs identified in the genome of the same tissue samples was performed using two distinct approaches, as previously described [50]: 1. Mapping of the miRNAs at the cytobands with high levels of CNAs and further selection based on their concordance level (i.e., cytoband with gains/amplifications of copy number/up-regulated miRNA expression and/or losses/deletions of copy number/down-regulated miRNA expression). Only the significant DNA segments affected by CNAs that were present in more than 25% of the cases (to assure that the CNAs were non-random and recurrent and were representative of most of the cases analysed), as identified in the aberration interval base reports (Agilent Cytogenomics v.3.0) were considered in this analysis. The location of each miRNA was determined using miRBase (http://www.mirbase.org); 2. Identification of common gene targets of the selected miRNAs above, that may be affected by both CNAs and miRNA expression alterations. In this second approach, for the previously selected miRNAs, gene targets were queried using the available miRNA target databases (Diana micro-T-CDS v.5.0 (diana. imis. athena-innovation/gr/DianaTools/index. phpr = microT_CDS/index) [114], miRDB (http://www.mirdb.org/miRDB/) [115] and TargetScan Release 7.1 (http://www.targetscan.org/vert_71/) [116]; only miRNA target genes that were present in two out of the three miRNA databases were selected.

### Biological function and pathway analysis

In order to assess the potential impact of the deregulated miRNAs identified above in cancer associated biological processes and pathways, Diana miRPath v.3.0 was used (http://snf-515788.vm.okeanos.grnet.gr/) based on adjusted *p*-values (FDR correction) [117]. Enrichment analysis of multiple miRNA gene targets comparing each set of miRNA targets to all known KEGG (Kyoto Encyclopedia of Genes and Genomes) pathways was obtained and selected by significant *p*-value (*p* < 0.05) and cancer-associated biological functions. CyKEGG Parser [118], GENEMANIA [119] and CyTargetLinker [120] (Cytoscape 3.5.1 software Applications [121]) were used to build KEGG pathways including miRNAs and their respective gene targets.

### Receiver operating characteristic (ROC) curve analysis

Receiver operating characteristic (ROC) curves and their area under the curve (AUC), was used to identify the power of the selected miRNAs in discriminating the TNBC and non-TNBC subtypes of the Latina patients. Sensitivity was plotted against 1-specificity for the binary classifier (TNBC and non-TNBC). An AUC of 100% denotes perfect discrimination by the miRNA, whereas an AUC of 50% denotes complete lack of discrimination by the miRNA. AUCs and 95% corresponding confidence intervals were calculated for each miRNA and for the combined panel of 17 miRNAs.

### Association analysis of clinical-pathological variables and the array-CGH and miRNA data

The Student *t* test with the unequal variance assumption was used to assess the differences in the mean age at diagnosis of the patients and mean tumor size in the TNBC and non-TNBC groups of patients. Fisher’s exact test was used to compare tumor stage, tumor grade, lymph node status and histology between the TNBC and non-TNBC groups. Discrete categories were grouped prior to the analysis as follows: For grade, category “I or II” included 26 samples and category “III” included 15 samples; for stage, category “moderately or well differentiated” included 26 samples and category “poorly differentiated” included 15 samples. A significance level of 0.05 was used for all these binary comparisons.

For the miRNAs that were selected from the integration with the array-CGH data, linear regression models were considered having the log-transformed miRNA values as the outcomes and tumor subtype, age, pathology, lymph node status, tumor grade or stage, and tumor size. Given the presence of cases with missing clinical-pathological data, both complete case analyses and multiple imputations to impute missing variables (with 10 imputed datasets) were performed using the aregImpute function in the Hmisc package version 4.1-1 in R [122, 123]. A significance level of FDR < 0.05 using the Benjamini and Hochberg FDR control method [124] was considered.

### Kaplan–Meier plot analysis

The KM Plotter Tool (http://kmplot.com/analysis/) was used to calculate hazard ratios, confidence intervals, and log-rank *P* values for each of the selected 17 miRNAs in relation to survival in the aggregated breast cancer clinical studies extracted from The Cancer Genome Atlas (TCGA) and Molecular Taxonomy of Breast Cancer International Consortium (METABRIC) databases (selected specifically for the TNBC subtype).

## SUPPLEMENTARY MATERIALS






