# Supplementary material for: Integrated copy number and miRNA expression analysis in triple negative breast cancer of Latin American patients
Source: Oncotarget. 2019 Oct 22;10(58):6184–203. doi: 10.18632/oncotarget.27250 (PMC6817452; doi:10.18632/oncotarget.27250)
Supplement: Supplementary file 1 [file oncotarget-10-6184-s001.pdf]

Integrated copy number and miRNA expression analysis in triple negative breast cancer of Latin American patients

SUPPLEMENTARY MATERIALS

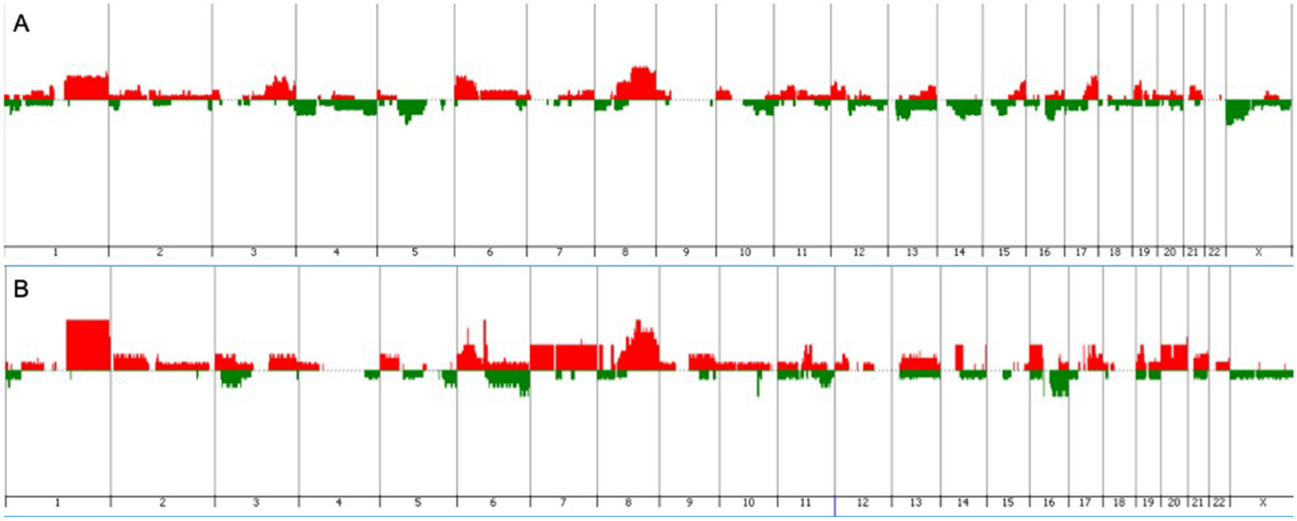

Supplementary Figure 1: Genomic view/penetrance plot of the array-CGH profiling of the 25 TNBC (**A**) and 16 non-TNBC (**B**) cases analyzed by array-CGH (Agilent Cytogenomcis v.3.0) showing the copy number alterations (CNAs). Vertical lines represent each chromosome number. Red peaks indicate copy number gains and green peaks indicate copy number losses.

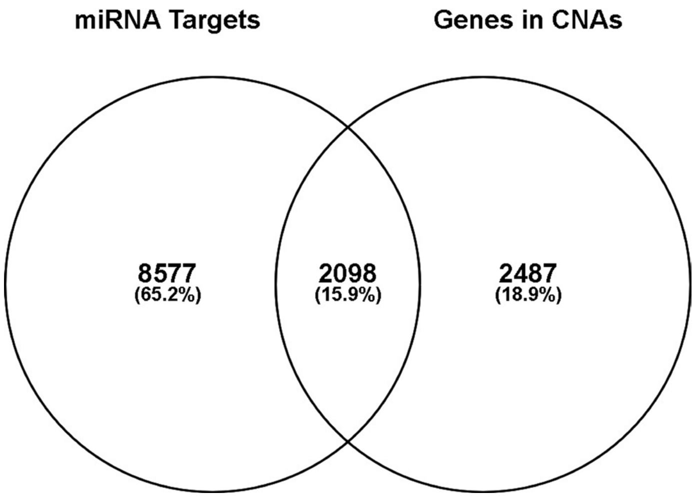

Supplementary Figure 2: Venn diagram showing the integration of genes located at the cytobands affected by CNAs and the corresponding gene targets of miRNAs mapped in these regions. (VENNY 2.1).

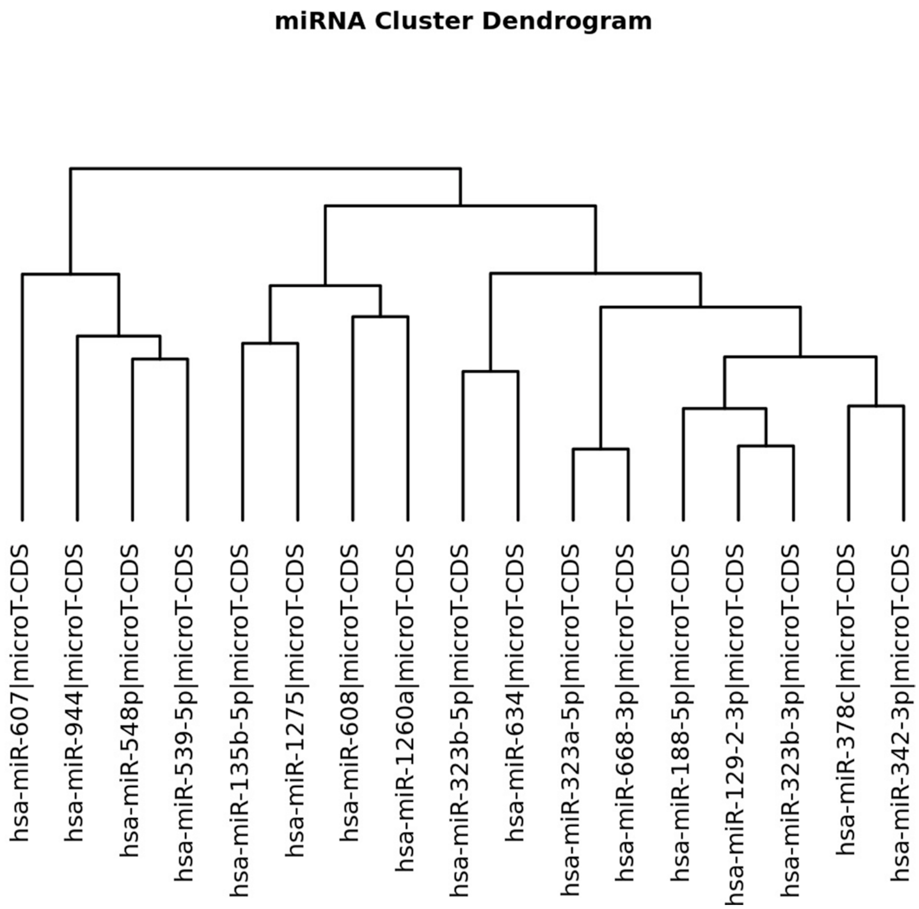

Supplementary Figure 3: MiRNA dendogram of the 17 miRNAs panel, based on miRNA-miRNA clustering (Diana miRpath, $p < 0.05$, FDR < 0.05.

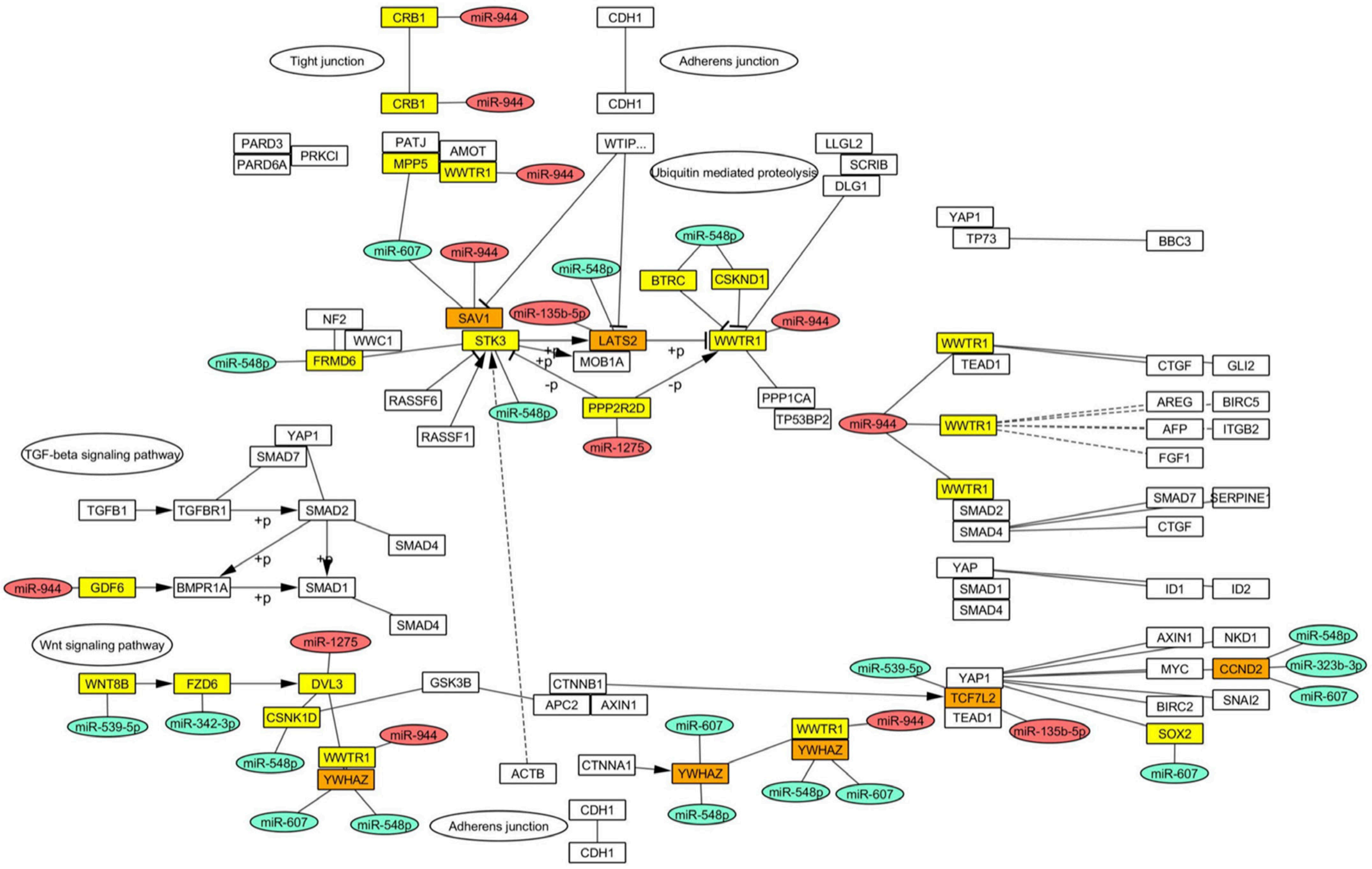

Supplementary Figure 4: Hippo signaling pathway showing 8 out of the 17 miRNAs panel and corresponding gene targets. In yellow: genes targeted by one miRNA; in orange: genes targeted by more than one miRNA; in green: down-regulated miRNAs, in red: up-regulated miRNAs (Cytoscape 3.5.1).

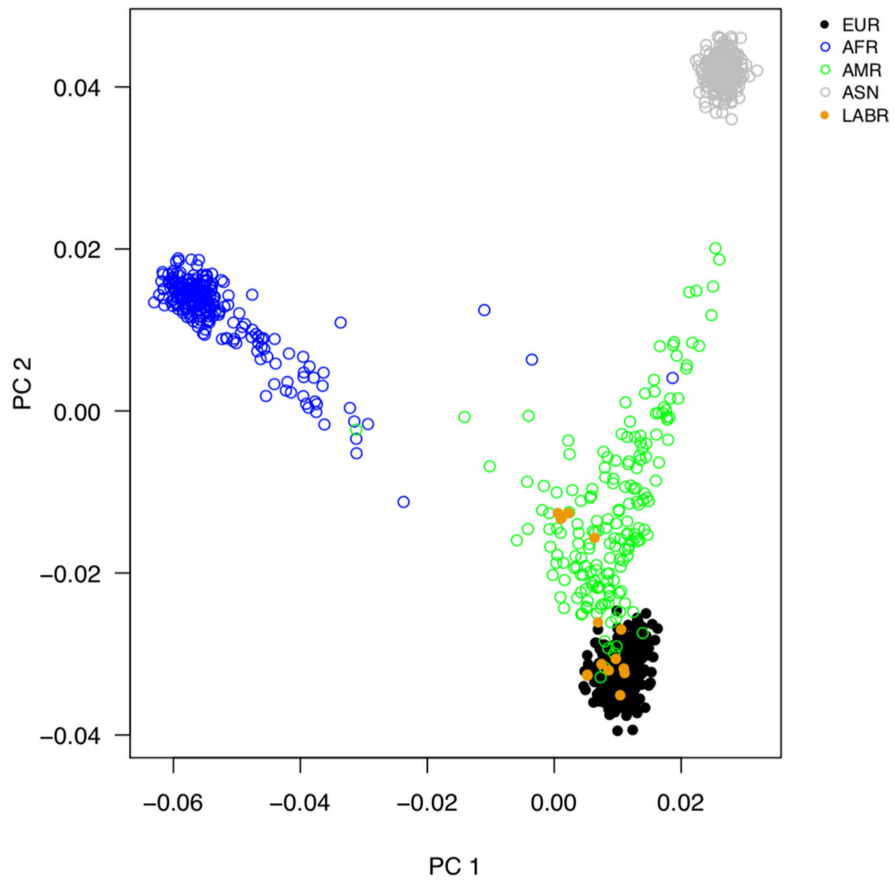

Supplementary Figure 5: Principal Component Analysis (PCA) showing the clustering of the Latina patients (orange dots) with the EUR and AMR groups based on genotype analysis (PLINK v. 1.9).

Supplementary Table 1: The 163 differentially expressed miRNAs among the TNBC and non-TNBC groups of the Latina patients´studied (presented by adj *p*-value). See Supplementary Table 1

Supplementary Table 2: Top 15 miRNAs observed up and down-regulated in the TNBC group of patients compared to the non-TNBC group (presented by Log2FC value)

miRNA	Log2FC	*p*	miRNA	Log2FC	*p*
hsa-miR-187-3p	3.64	0.009	hsa-miR-720	−3.98	0.012
hsa-miR-601	2.68	0.02	hsa-miR-1260a	−3.30	0.004
hsa-miR-663a	2.61	0.006	hsa-miR-4286	−3.09	0.008
hsa-miR-421	2.28	0.005	hsa-miR-4454	−2.72	0.047
hsa-miR-378b	2.26	0.003	hsa-miR-200c-3p	−2.45	0.013
hsa-miR-1305	2.22	0.019	hsa-let-7b-5p	−2.24	0.029
hsa-miR-135b-5p	1.93	0.001	hsa-miR-199a-3p	−2.19	0.032
hsa-miR-638	1.90	0.001	hsa-miR-199b-3p	−2.19	0.032
hsa-miR-708-5p	1.87	0.005	hsa-let-7c	−2.19	0.007
hsa-miR-1290	1.83	0.021	hsa-let-7a-5p	−2.09	0.041
hsa-miR-548z	1.68	0.036	hsa-miR-125b-5p	−1.87	0.023
hsa-miR-2117	1.63	0.002	hsa-miR-26a-5p	−1.85	0.034
hsa-miR-567	1.63	<0.05	hsa-let-7e-5p	−1.80	0.028
hsa-miR-500b	1.60	0.01	hsa-miR-125a-5p	−1.73	0.048
hsa-miR-526a	1.55	0.005	hsa-miR-193a-5p	−1.72	0.007

Supplementary Table 3: The 38 KEGG pathways affected by the most significant 100 differentially expressed miRNAs among the TNBC and non-TNBC groups (presented by adj *p*-value)

#	KEGG pathway	adj *p*-value	#genes	#miRNAs
1	ECM-receptor interaction	4.16E-06	65	66
2	Adherens junction	4.16E-06	63	70
3	Mucin type O-Glycan biosynthesis	7.27E-06	25	33
4	Morphine addiction	1.34E-05	71	68
5	Proteoglycans in cancer	1.34E-05	148	78
6	Arrhythmogenic right ventricular cardiomyopathy (ARVC)	4.15E-05	56	65
7	GABAergic synapse	8.07E-05	66	67
8	Hippo signaling pathway	0.000422	110	69
9	Endocytosis	0.000937	153	78
10	Ras signaling pathway	0.001279	164	86
11	ErbB signaling pathway	0.001337	69	75
12	Rap1 signaling pathway	0.00197	155	82
13	PI3K-Akt signaling pathway	0.002122	245	86
14	Pathways in cancer	0.002122	288	87
15	Glutamatergic synapse	0.002125	86	67
16	Nicotine addiction	0.00534	30	54
17	Lysine degradation	0.00534	37	64
18	Thyroid hormone signaling pathway	0.00534	88	74
19	Focal adhesion	0.00534	152	79
20	mTOR signaling pathway	0.005391	49	67
21	Circadian rhythm	0.005785	27	57
22	Central carbon metabolism in cancer	0.011361	50	64
23	Retrograde endocannabinoid signaling	0.011361	76	69
24	TGF-beta signaling pathway	0.011361	57	74
25	Circadian entrainment	0.012166	73	76
26	Amoebiasis	0.014985	78	69
27	Renal cell carcinoma	0.018511	50	70
28	Wnt signaling pathway	0.018918	104	77
29	Pancreatic cancer	0.020395	49	70
30	Axon guidance	0.02064	91	74
31	FoxO signaling pathway	0.021891	98	73
32	Long-term depression	0.02446	44	58
33	Thyroid hormone synthesis	0.02446	52	60
34	Oxytocin signaling pathway	0.036428	113	86
35	Colorectal cancer	0.038055	48	66
36	Signaling pathways regulating pluripotency of stem cells	0.041672	100	76
37	Platelet activation	0.043618	92	70
38	Phosphatidylinositol signaling system	0.047876	56	73

Supplementary Table 4: Top 10 KEGG pathways potentially affected by the 17 miRNAs. See Supplementary Table 4

Supplementary Table 5: KEGG pathways potentially affected by 10 out of the 17 miRNAs identified and their selected gene targets (DIANA mirPath 3.0)

Glycosaminoglycan biosynthesis – heparan sulfate / heparin (hsa00534, p = 0,01967)	
miR-135b-5p	*EXT1*
miR-944	*EXT1*
miR-342-3p	*EXT1*
miR-1260a	*EXT2*
miR-539-5p	*EXT2*
Biosynthesis of unsaturated fatty acids (hsa01040, p = 0,01967)	
miR-548p	*ACOX1*
miR-135b-5p	*ELOVL2*
miR-1275	*ELOVL2*
miR-129-2-3p	*ELOVL2*
miR-944	*HSD17B12, SCD*
Hippo signaling pathway (hsa04390, p = 0,01967)	
miR-1275	*DVL3, PPP2RD2*
miR-135b-5p	*LATS2, TCF7L2*
miR-323b-3p	*CCND2*
miR-342-3p	*FZD6*
miR-539-5p	*TCF7L2, WNT8B*
miR-548p	*BTRC, CCND2, CSNK1D, FRMD6, LATS2, STK3, YWHAZ*
miR-607	*CCND2, MPP5, SAV1, SOX2, YWHAZ*
miR-944	*CRB1, GDF6, SAV1, WWTR1*

Supplementary Table 6: Kaplan–Meier test results of eight out of the panel of 17 miRNAs that were differentially expressed in the TNBC and non-TNBC dataset of the METABRIC and TCGA data (KMPlot - miRpower)

miRNA	HR	IC 95%	*P*	Database
hsa-miR-135b-5p	1.59	1.01–2.5	0.041	METABRIC
hsa-miR-323b-3p	0.62	0.4–0.97	0.035	METABRIC
hsa-miR-548p	0.25	0.08–0.76	0.0086	TCGA
hsa-miR-607	0.2	0.07–0.59	0.0014	TCGA
hsa-miR-608	0.25	0.08–0.76	0.0086	TCGA
hsa-miR-634	1.85	1.02–3.35	0.04	METABRIC
hsa-miR-668	0.2	0.07–0.63	0.0023	TCGA
hsa-miR-1260a	0.25	0.08–0.76	0.0086	TCGA
