# Supplementary material for: Integrated copy number and miRNA expression analysis in triple negative breast cancer of Latin American patients
Source: Oncotarget. 2019 Oct 22;10(58):6184–203. doi: 10.18632/oncotarget.27250 (PMC6817452; doi:10.18632/oncotarget.27250)
Supplement: Supplementary file 2 [file oncotarget-10-6184-s002.pdf]

Supplementary TABLE 1: The 163 differentially expressed miRNAs among the TNBC and non-TNBC groups of the Latina patients´studied (presented by adj p-value).

miRNAs	Log2FC	adj p-value	miRNAs	Log2FC	adj p-value
hsa-miR-1299	1.241552	0.001	hsa-miR-548u	-0.50615	0.009
hsa-miR-135b-5p	1.928202	0.001	hsa-miR-577	0.968527	0.009
hsa-miR-3151	-0.56665	0.001	hsa-miR-1200	1.019496	0.01
hsa-miR-323a-5p	-0.59037	0.001	hsa-miR-184	-0.5105	0.01
hsa-miR-412	1.272243	0.001	hsa-miR-500b	1.601524	0.01
hsa-miR-555	-0.59636	0.001	hsa-miR-936	1.068307	0.01
hsa-miR-638	1.897654	0.001	hsa-miR-518c-3p	0.985269	0.011
hsa-miR-2117	1.634233	0.002	hsa-miR-943	-0.45362	0.011
hsa-miR-298	-0.6011	0.002	hsa-miR-1229	-0.43502	0.012
hsa-miR-299-3p	1.171332	0.002	hsa-miR-139-5p	-0.49376	0.012
hsa-miR-496	1.133951	0.002	hsa-miR-219-2-3p	-0.42312	0.012
hsa-miR-499b-5p	-0.56337	0.002	hsa-miR-3161	1.043253	0.012
hsa-miR-518a-3p	0.804529	0.002	hsa-miR-599	-0.516	0.012
hsa-miR-548p	-0.53097	0.002	hsa-miR-671-3p	-0.46463	0.012
hsa-miR-556-3p	-0.52457	0.002	hsa-miR-720	-3.97743	0.012
hsa-miR-610	0.811232	0.002	hsa-miR-200c-3p	-2.44742	0.013
hsa-miR-650	-0.55002	0.002	hsa-miR-323a-3p	0.628786	0.013
hsa-miR-378b	2.259158	0.003	hsa-miR-519b-5p	0.78908	0.013
hsa-miR-765	-0.56628	0.003	hsa-miR-519c-5p	0.78908	0.013
hsa-miR-1260a	-3.30145	0.004	hsa-miR-663b	-1.3021	0.013
hsa-miR-1286	1.241175	0.004	hsa-miR-1197	0.727561	0.014
hsa-miR-219-5p	1.167238	0.004	hsa-miR-548k	0.790092	0.014
hsa-miR-378g	1.050617	0.004	hsa-miR-563	-0.67788	0.014
hsa-miR-568	0.924021	0.004	hsa-miR-668	-0.44523	0.014
hsa-miR-802	0.959188	0.004	hsa-miR-762	1.056555	0.014
hsa-miR-944	1.133203	0.004	hsa-miR-1227	-0.45401	0.015
hsa-miR-1263	-0.47719	0.005	hsa-miR-637	-0.47987	0.015
hsa-miR-2276	0.826868	0.005	hsa-miR-378f	1.416524	0.016
hsa-miR-421	2.280377	0.005	hsa-miR-589-5p	-0.43799	0.016
hsa-miR-513a-3p	1.148336	0.005	hsa-miR-140-3p	-0.49958	0.017
hsa-miR-518d-5p	1.550398	0.005	hsa-miR-3144-5p	-0.47359	0.017
hsa-miR-520c-5p	1.550398	0.005	hsa-miR-379-5p	1.537607	0.017
hsa-miR-526a	1.550398	0.005	hsa-miR-514a-3p	0.70441	0.017
hsa-miR-634	0.903045	0.005	hsa-miR-876-5p	-0.43982	0.017
hsa-miR-708-5p	1.866813	0.005	hsa-miR-378e	-1.59139	0.018
hsa-miR-1269a	0.860137	0.006	hsa-miR-1305	2.21868	0.019
hsa-miR-1322	1.258168	0.006	hsa-miR-499a-3p	0.766943	0.019
hsa-miR-4425	-0.55469	0.006	hsa-miR-1296	-0.43391	0.02
hsa-miR-607	-0.4984	0.006	hsa-miR-188-5p	-0.92524	0.02
hsa-miR-620	-0.53163	0.006	hsa-miR-601	2.680344	0.02
hsa-miR-663a	2.60688	0.006	hsa-miR-1181	0.672413	0.021
hsa-let-7c	-2.18788	0.007	hsa-miR-1276	1.37538	0.021
hsa-miR-193a-5p	-1.71617	0.007	hsa-miR-1290	1.825019	0.021
hsa-miR-125a-3p	1.015675	0.008	hsa-miR-3147	-1.23049	0.021
hsa-miR-1910	-0.48273	0.008	hsa-miR-4792	0.60057	0.022
hsa-miR-323b-3p	-0.49473	0.008	hsa-miR-96-5p	0.875759	0.022

TABLE S1: cont.

miRNAs	Log2FC	adj p-value	miRNAs	Log2FC	adj p-value
hsa-miR-4286	-3.08769	0.008	hsa-miR-125b-5p	-1.87456	0.023
hsa-miR-505-3p	0.90293	0.008	hsa-miR-18a-5p	1.053774	0.024
hsa-miR-576-5p	0.659678	0.008	hsa-miR-378c	-0.42007	0.024
hsa-miR-187-3p	3.642798	0.009	hsa-miR-519b-3p	-0.57555	0.024
hsa-miR-323b-5p	-0.48649	0.009	hsa-miR-302d-3p	-0.50028	0.026
hsa-miR-548s	-0.73219	0.026	hsa-miR-1207-3p	-0.45121	0.041
hsa-miR-887	-0.44763	0.027	hsa-miR-1303	1.250427	0.041
hsa-let-7e-5p	-1.79934	0.028	hsa-miR-34c-5p	0.487047	0.041
hsa-miR-769-5p	1.013276	0.028	hsa-miR-513b	-0.46875	0.041
hsa-let-7b-5p	-2.24155	0.029	hsa-miR-1185-5p	0.689035	0.042
hsa-miR-1260b	0.788523	0.03	hsa-miR-300	0.509251	0.043
hsa-miR-548ak	0.724235	0.03	hsa-miR-19b-3p	1.210221	0.044
hsa-miR-1291	-0.43629	0.031	hsa-miR-324-3p	0.615431	0.045
hsa-miR-3182	-0.96625	0.031	hsa-miR-30c-5p	1.250616	0.046
hsa-miR-199a-3p	-2.19484	0.032	hsa-miR-593-3p	0.964414	0.046
hsa-miR-199b-3p	-2.19484	0.032	hsa-miR-760	0.45456	0.046
hsa-miR-367-3p	1.153571	0.032	hsa-miR-1178	-1.06657	0.047
hsa-miR-410	0.743609	0.033	hsa-miR-3190-5p	0.457707	0.047
hsa-miR-1277-3p	0.873712	0.034	hsa-miR-4454	-2.72352	0.047
hsa-miR-1284	0.660131	0.034	hsa-miR-125a-5p	-1.72745	0.048
hsa-miR-26a-5p	-1.85397	0.034	hsa-miR-571	-0.3322	0.048
hsa-miR-29c-3p	-1.54592	0.034	hsa-miR-1233	0.963333	0.049
hsa-miR-2052	0.704888	0.035	hsa-miR-1268a	1.379848	0.049
hsa-miR-129-2-3p	0.76581	0.036	hsa-miR-1275	0.610095	0.049
hsa-miR-370	0.620461	0.036	hsa-let-7d-5p	-1.28678	0.05
hsa-miR-548z	1.675082	0.036	hsa-miR-1273g-5p	-0.38748	0.05
hsa-miR-550a-5p	0.806703	0.036	hsa-miR-147b	0.502335	0.05
hsa-miR-191-5p	-1.683	0.037	hsa-miR-450b-5p	0.648325	0.05
hsa-miR-378d	-0.34324	0.037	hsa-miR-1225-5p	1.160807	<0.05
hsa-miR-4741	0.952086	0.038	hsa-miR-1258	1.331858	<0.05
hsa-miR-1912	-0.45077	0.039	hsa-miR-1302	-0.56988	<0.05
hsa-miR-342-3p	-1.50766	0.039	hsa-miR-488-3p	-0.62421	<0.05
hsa-miR-520e	1.140733	0.039	hsa-miR-539-5p	-0.68292	<0.05
hsa-miR-608	-0.52486	0.04	hsa-miR-567	1.63399	<0.05
hsa-let-7a-5p	-2.09134	0.041	hsa-miR-933	-0.56734	<0.05
hsa-miR-1203	-0.41278	0.041			
