# Supplementary material for: Integrated copy number and miRNA expression analysis in triple negative breast cancer of Latin American patients
Source: Oncotarget. 2019 Oct 22;10(58):6184–203. doi: 10.18632/oncotarget.27250 (PMC6817452; doi:10.18632/oncotarget.27250)
Supplement: Supplementary file 3 [file oncotarget-10-6184-s003.pdf]

Supplementary TABLE 4. Top 10 KEGG pathways potentially affected by the 17 miRNAs.

KEGG pathway	adj p-value	#miRNAs	miRNAs	#genes	putative target genes
Axon guidance (hsa04360)	3.81E-08	17	miR-135b-5p, miR-944, miR-548p, miR-1275, miR-607, miR-608, miR-378c,miR-129-2-3p, miR-1260a, miR-342-3p, miR-323a-5p, miR-539-5p, miR-668, miR-323b-3p, miR-323b-5p, miR-634, miR-188-5p	66	*SEMA3G, SEMA6A, GSK3B, ABLIM3, PLXNB2, NTNG2, MET, ITGB1, ROCK1, SEMA4A, RAC2, CXCR4, NRAS, PAK2, ARHGEF12, EPHA5, ROCK2, PAK7, ROBO2, PPP3R1, SRGAP1, NTNG1, PLXNA1, GNAI3, RHPA, KRAS, PAK3, FYN, EFNA5, EPHA7, SEMA4G, NCK1, PPP3CA, PTK2, RASA1, EFNB3, SLIT2, DCC, PPP3CB, SRGAP3, EPHA3, DPYSL2, UNC5C, CFL2, SEMA3D, NRP1, RAC1, PLXNB1, SEMA4B, UNC5B, SEMA3A, PAK6, EDNB1, PLXNC1, LIMK1, SEMA4D, ROBO1, EPHA4, SEMA3E, UNC5D, LRRC4, EPHB1, GNAI1, PPPR32*
Glycosaminoglycan biosynthesis - chondroitin sulfate / dermatan sulfate (hsa00532)	1.57E-07	7	miR-135b-5p, miR-944, miR-1275,miR-548p, miR129-2-3p, miR342-3p, miR539-5p,	12	*UST, CHST3, CHST15, CHST11, CHSY3, DSE, CHPF, CHST14, CSGALNACT1, CHSY1, XYLT1*
Mucin type O-Glycan biosynthesis (hsa00512)	9.39E-06	5	miR-944,miR-548p,miR-607,miR-539-5p,miR-342-3p	14	*POC1B-GALNT4, GALNT7, GALNT15, GALNTL5, GALNT13, GALNT4, GCNT3, GALNT1, GALNT10, CIGALT1, GCNT1, CIGALT1C1, GALNT5, GALNT16*
Thyroid hormone signaling pathway (hsa04919)	9.39E-06	15	miR-135b-5p, miR-944, miR-548p, miR-607, miR-608, miR-378c, miR-129-2-3p, miR-1260a, miR-342-3p, miR-323a-5p, miR-539-5p, miR-668, miR-323b-5p, miR-634, miR-1275	57	*ESR1, ACTB, GSK3B, PRKCA, ATP1B2, MED13L, NRAS, MED14, DIO2, PIK3R2, MED13, SLC16A10, ATP1B1, THRA, MED17, RCAN2, KRAS, MED12L, MED1, MED4, ITGAV, MED24, PLCB1, TBC1D4, PIK3CD, PIK3R3, HIF1A, PLN, PRKACA, PRKCG, NCOR1, ATP1A4, NOCA2, PIK3R1, PIK3CG, HDAC2, PRKX, PLCG2, THRB, KAT2B, EP300, AKT3, SLC2+A3, PLCE1, PIK3CA, SLC2A1, PDPK1, PFKFB2, ATP2A2, FOXO1, MDM2, PLCB4, BMP4, NCOA1, PRKACB, RXRA, PIK3R2*

TABLE S4. cont

KEGG pathway	adj p-value	#miRNAs	miRNAs	#genes	*putative target genes*
Signaling pathways regulating pluripotency of stem cells	2.32E-05	16	miR-135b-5p, miR-944,miR-548p, miR-1275, miR-607, miR-608, miR-378c, miR-129-2-3p, miR-1260a, miR-342-3p, miR-323a-5p, miR-539-5p, miR-668, miR-323b-3p, miR-323b-5p, miR-634	68	*BMI1, GSK3B, DVL3, FZD5, OTX1, KAT6A, PAX6, SMAD2, NRAS, NODAL, INHBB, APC, REST, ACVR1B, WNT5A, PIK3R2, MAPK14, TBX3, FZD6, SMARCAAD1, WNT2B, INHBA, WNT3, IGF1R, ZFHX3, ID4, FZD8, KRAS, FZD3, POU5F1B, ACVR2B, PCGF5, FZD4, RIF1, PIK3CD, PIK3R3, FZD10, JAK2, AXIN2, LIFR, SKIL, ZIC3, SMAD5, PIK3R1, PIK3CG, ACVR2A, FGF2, BMP2, FZD1, ACVR1C, IGF1, AKT3, BMPR1A, PIK3CA, WNT8B, IL6ST, ISL1, FGFR1, SOX2, PCGF3, WNT7B, KLF4, JAK1, BMP4, MEIS1, BMPR2, PIK3R2*
Proteoglycans in cancer (hsa05205)	2.46E-05	16	miR-135b-5p, miR-944,miR-548p, miR-1275, miR-607, miR-608, miR-378c,miR-129-2-3p, miR-342-3p, miR-323a-5p, miR-539-5p, miR-668,miR-323b-3p, miR-323b-5p, miR-634, miR-188-5p	88	*ESR1, CAMK2D, BRAF, ACTB, PRKCA, FZD5, MET, ITGB1, ROCK1, SMAD2, CBL, NRAS, THBS1, PTCH1, WNT5A, PIK3R2, ARHGEF12, PPP1CC, MAPK14, ROCK2, FRS2, FZD6, RDX, IQGAP1, ITGA5, WNT2B, TIAM1, WNT3, IGF1R, EGFR, RHOA, PPP1R12B, CAV2, FZD8, KRAS, FZD3, RRAS2, MSN, ANK2, CTTN, CAMK2A, PTK2, CBLB, ITGAV, ANK3, FZD4, PPP1R12A, PIK3CD, PIK3R3, FZD10, GPC1, HIF1A, IGF2, FLNB, PRKACA, PRKCG, EIF4B, TIMP3, FLNA, ITGA2, PIK3R1, SOS1, PIK3CG, PTPN11, PRKX, DDX5, RAC1, FGF2, PLCG2, PPP1R12C, FZD1, IGF1, AKT3, PLCE1, PIK3CA, WNT8B, SMP, PDPK1, VEGFA, FGFR1, ITPR2, WNT7B, MDM2, ERBB4, RPS6KB1, PRKACB, PPP1CB, PIK3R2*
Wnt signaling pathway (hsa04310)	3.14E-05	17	miR-135b-5p, miR-944, miR-548p, miR-1275, miR-607, miR-608, miR-378c, miR-129-2-3p, miR-1260a, miR-342-3p, miR-323a-5p, miR-539-5p, miR-668, miR-323b-3p, miR-323b-5p, miR-634, miR-188-5p	66	*CAMK2D, GSK3B, PRKCA, DVL3, FZD5, LRP6, RAC2, BTRC, APC, VANGL1, TCF7L2, WNT5A, CCND2, ROCK2, FZD6, PPP3R1, CTBP2, WNT2B, WNT3, LRP5, RHOA, FZD8, FZD3, SKP1, FRAT2, CAMK2A, PPP3CA, PRICKLE1, NLK, PLCB1, FZD4, SENP2, FZD10, PPP3CB, AXIN2, MAPK8, CSNK1A1, NKD2, PRKACA, VANGL2, PRKCG, CSNK2B, CSNK2A1, PRKX, RAC1, SIAH1, PSEN1, FZD1, PRICKLE2, CSNK1E, EP300, CXXC4, RBX1, WNT8BM LEF1, DAAM1, MAP3K7, FBXW11, NFATC1, WNT7B, TLB1XR1, PPARD, PLCB4,*

TABLE S4. cont

KEGG pathway	adj p-value	#miRNAs	miRNAs	#genes	*putative target genes*
Hippo signaling pathway (hsa04390)	7.29E-05	15	miR-135b-5p, miR-944, miR-548p, miR-1275, miR-607, miR-608, miR-378c, miR-129-2-3p, miR-1260a, miR-342-3p, miR-323a-5p, miR-539-5p, miR-668, miR-323b-3p, miR-634	67	*ACTB, GSK3B, DVL3, FZD5, TGFBR1, YWHAH, YAP1, SMAD2, YWHAE, BTRC, APC, TCF7L2, WNT5A, YWHAG, PPP1CC, CCND2, BMP5, FLI2, AREG, FZD6, WNT2B, MOB1B, WANT3, CRB1, PPP2REB, PPP2R2D, WWTR1, FZD8, TP53BP2, FZD3, BMP8B, LIMD1, MPP5, FZD4, CSNK1D, DLG4, FZD10, AXIN2, PPP2R2A, RASSF6, PPP2R1A, FRMD6, SAV1, TEAD1, YWHAZ, GDF6, STK3, BMP2, FZD1, CSNK1E, BMPR1A, CTNNA3, WNT8, DLG2, LEF1, PARD3, CTNNA2, LATS1, SOX2, BMP7, LATS2, FBXW11, PARD6B, WNT7B, BMP4, BMPR2, PPP1CB*
Ras signaling pathway	0.0001079	17	miR-135b-5p, miR-944, miR-548p, miR-1275, miR-607, miR-608, miR-378c, miR-129-2-3p, miR-1260a, miR-342-3p, miR-323a-5p, miR-539-5p, miR-668, miR-323b-3p, miR-323b-5p, miR-634, miR-188-5p	95	*FGF12, PRKCA, KSR2, PDGFRA, GNG13, NFKB1, MET, FIGF, GNGT1, RAC2, NRAS, STK4, RASA2, CALM1, PAK2, PIK3R2, RAP1A, RASGRF2, ETS1, PAK7, GNG12, ANGPT2, RALA, CHUK, RALBP1, PLD1, TIAM1, RASGRP2, IGF1R, EGFR, RHOA, KRAS, PAK3, RRAS2, EFNA5, PLA2G2D, RAB5A, MLLT4, RASA1, PIK3CD, PIK3R3, PLA2G12A, RASGRP1, MAPK8, PLA2G16, REL, BRAP, PRKACA, PRKCG, FLT1, GNG2, PLA2G12B, PIK3R1, SOS1, PIK3CG, PTPN11, PRKX, RASA4, TBK1, KITLG, RAC1, FGF2, PLCG2, NF1, IGF1, SHC4, BCL2L1, AKT3, PDGFC, PAK6, ANGPT1, PLCE1, PDGFD, PIK3CA, SYNGAP1, GAB2, FGF8, GNG4, RASAL2, RGL1, TAB5C, FGFR2, VEGFA, FGFR1, ABL2, FGF7, CSF1, RAP1B, RAPGEF5, GNG3, MAPK10, PRKACB, PDGFA, GRIN2B, PIK3R2*

TABLE S4. cont

KEGG pathway	adj p-value	#miRNAs	miRNAs	#genes	*putative target genes*
Pathways in cancer (hsa05200)	0.0001787	17	miR-135b-5p, miR-944, miR-548p, miR-1275, miR-607, miR-608, miR-378c, miR-129-2-3p, miR-1260a, miR-342-3p, miR-323a-5p, miR-539-5p, miR-668, miR-323b-3p, miR-323b-5p, miR-634, miR-188-5p	169	*BRAF, FGF12, GSK3B, PRKCA, DVL3, PDGFRA, FZD5, E2F1, TGFBR1, GNG13, NFKB1, MET, ADCY1, ADCY5, SPI1, ITGB1, FIGF, PTGER4, ROCK1, GNGT1, CXCL8, RAC2, SMAD2, CBL, E2F2, CXCR4, ADCY7, NRAS, CRKL, STK4, LPAR3, APC, CRK, PTCH1, RAD51, MSH3, CUL2, TPR, TCF7L2, WNT5A, TGFA, HHIP, COL4A5, PIK3R2, ARHGEF12, GNA13, GLI2, ROCK2, ARNT, ETS1, ADCY3, GNG12, RALA, FZD6, CTBP2, CHUK, BCL2, CDKN1B, WNT2B, TRAF4, RALBP1, PLD1, CDKN2B, WNT3, RASGRP2, IGF1R, EGFR, GNAI3, PTCH2, F2RL3, RHOA, TRAF5, APPL1, FZD8, KRAS, CDK6, FZD3, PAX8, VHL, MITF, LPAR4, PTK2, GNA11, CBLB, ITGAV, PLCB1, FZD4, RUNX1T1, DCC, AR, PIK3CD, PIK3R3, FZD10, MSH6, AXIN2, RASGRP1, SKP2, HIF1A, E2F3, MAPK8, PTGER2, DAPK1, PRKACA, PRKCG, GNG2, COL4A3, ITGA2, PIK3R1, RB1, COL4A4, SOS1, PIK3CG, HDAC2, PRKX, KITLG, RAC1, FGF2, BMP2, PLCG2, FZD1, FLT3, IGF1, EP300, CASP8, GNAQ, NKX3-1, BCL2L1, AKT3, PIAS2, CTNNA3, PIK3CA, RBX1, WNT8B, LEF1, COL4A6, MECOM, FGF8, SMO, SLC2A1, GNG4, EDNRA, SUFU, FGFR2, ITGA6, SHH, CTNNA2, VEGFA, PTEN, FGFR1, FOXO1, FGF7, TRAF3, WNT7B, PPARD, JAK1, MDM2, PLCB4, BMP4, GNG3, CCDC6, MAPK10, GNAI1, PRKACB, XIAP, EGLN1, RXRA, PDGFA, LAMA4, PIK3R2*
